# Gene Expression Profiling of Pancreatic Ductal Adenocarcinoma Cells in Hypercapnia Identifies SIAH3 as a Novel Prognostic Biomarker

**DOI:** 10.3390/ijms26072848

**Published:** 2025-03-21

**Authors:** Nitzan Zohar, Ryan Maguire, Saed Khalilieh, Aditi Jain, Dmitriy Bosykh, Wilbur B. Bowne, Harish Lavu, Charles J. Yeo, Avinoam Nevler

**Affiliations:** Jefferson Pancreas, Biliary and Related Cancer Center, Department of Surgery, Thomas Jefferson University, 1015 Walnut Street, Philadelphia, PA 19107, USA; nitszohar@gmail.com (N.Z.); ryan.maguire@students.jefferson.edu (R.M.); saed.khalilieh@gmail.com (S.K.); aditi.jain@jefferson.edu (A.J.); dmitriy.bosykh@jefferson.edu (D.B.); wilbur.bowne@jefferson.edu (W.B.B.); harish.lavu@jefferson.edu (H.L.); charles.yeo@jefferson.edu (C.J.Y.)

**Keywords:** pancreatic ductal adenocarcinoma, hypercapnia, tumor microenvironment, SIAH3, prognostic marker

## Abstract

Hypercapnia is a key feature of the respiratory microenvironment in many pathologic conditions. It occurs both as a regional and as a systemic process, and it is associated with multiple metabolic changes such as mitochondrial dysfunction, decreased ATP production, and metabolic shift from glycolytic energy production to fatty acid metabolism. In the cancer tumor microenvironment, hypercapnia has been linked at times to enhanced cell migration, invasion, and chemoresistance. Our previous work has shown that hypercapnia-associated gene signatures can be used as prognostic biomarkers. However, unlike the hypoxia-inducible factor pathway, there are no validated targets to quantify hypercapnia. In this study, we investigated the phenotypic and transcriptomic changes occurring in pancreatic ductal adenocarcinoma (PDAC) due to chronic exposure to hypercapnic atmospheres. We then identified and validated SIAH3 as a hypercapnia-affected target and explored its clinical relevance as a prognostic factor in PDAC.

## 1. Introduction

Multiple cancer types are characterized by hypercapnic microenvironments [1,2], often concomitantly presenting with hypoxia as well. However, relative to hypoxia, hypercapnia has received limited attention in the study of the respiratory tumor microenvironment (TME). While the hypercapnia signaling pathway is not well understood and cellular hypercapnia sensors have yet to be identified, previous research has indicated that hypercapnic TME increases invasiveness and growth of colorectal cancer cells [3,4] and induces platinum chemoresistance in lung cancer cells [2]. We previously reported that hypercapnia induces both increased replication in pancreatic cancer cells and platinum chemoresistance [5]. Furthermore, we also reported that hypercapnic gene expression patterns were strong prognostic markers for survival and disease progression in patients with pancreatic ductal adenocarcinoma (PDAC) [6].

Physiologic studies of hypercapnia in non-cancer models have shown hypercapnia to result in varied effects across different tissues [7]. While it is usually considered to be an inhibitory factor of cell growth, several studies have noted it to be associated with accelerated wound healing and increased tissue blood flow [8,9,10,11,12]. Tsuji et al. [10] showed hypercapnia to promote proliferation in human umbilical vein endothelial cells (HUVEC) and even abrogate the slowed replication induced by hypoxia (1% O_2_). Hypercapnia appears to be closely associated with the regulation of metabolic functions as it has been shown to interfere with mitochondrial activity, decrease mitochondrial ATP production [13], shift the metabolism from glycolysis to fatty acid-based energy production [14,15], and activate cholesterol production pathways [16].

Physiologic studies in murine lungs and muscles have previously revealed hypercapnia to promote extracellular matrix remodeling in neonatal mouse lungs [17,18] and negatively regulate the β-catenin signaling pathway [19]. Gene expression analyses in mouse tissues exposed to acute and chronic hypercapnia revealed regulation of multiple key cellular transcription factors, such as hypoxia-inducible factor 1α (HIF1α), c-Myc, CREB1, estrogen receptor 1 (ESR1), androgen receptor (AR), and TP53 [7].

Though there is limited clinical cancer data regarding the specific impact of hypercapnia on cancer cells, several studies have drawn a link between elevated systemic levels of carbon dioxide and worse oncological outcomes [20,21].

In this study, we aimed to explore the phenotype and gene expression pattern in PDAC pancreatic cancer cells exposed to chronic hypercapnia and identify potential novel targets.

## 2. Results

### 2.1. Chronic Hypercapnia Exposure Induces a Distinct Aggressive Phenotype in Pancreatic Cancer Cells

**Figure 1:**
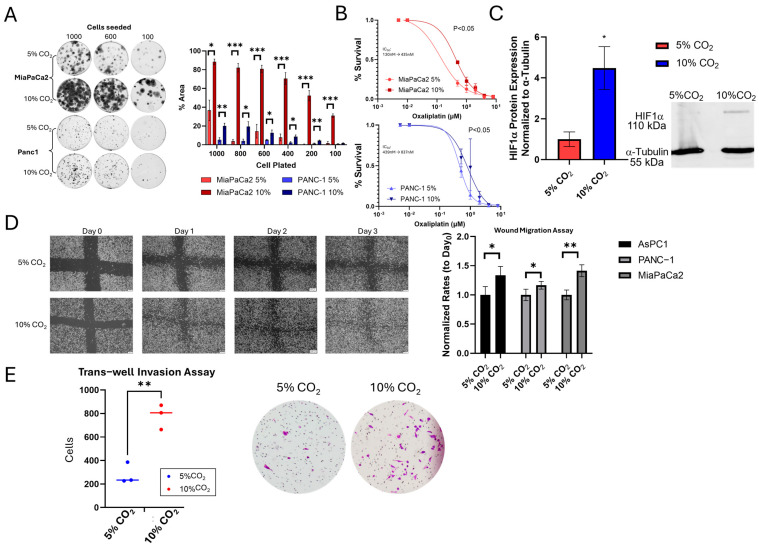
Chronic exposure to hypercapnia induces an aggressive phenotype in pancreatic ductal adenocarcinoma cells (PDAC): in vitro effect of chronic hypercapnia exposure (10% CO_2_) compared to normocapnia (5% CO_2_). (**A**) Colony formation assays in 2 cell lines (MiaPaCa2, Panc1) assessing multiple cell plating densities, showing increased growth in hypercapnia (N = 3) with representative images (**left**) and quantification (**right**). (**B**) Cell cytotoxicity assessment of oxaliplatin, showing increased therapy resistance in chronic hypercapnia (N = 3). (**C**) Hypoxia-inducible Factor-1α (HIF1α) protein expression is increased in chronic hypercapnia exposure (MiaPaCa2 cells, N = 3). (**D**) Wound scratch assay (N = 6) showing that chronic hypercapnia promotes PDAC cell migration (MiaPaCa2, Panc1, and AsPC-1). The left panel has representative images from ASPC1 cells; the right panel has quantification. (**E**) Transwell migration assay (N = 3) showing chronic hypercapnia is linked with greater cell invasion in MiaPaCa2 cells. * *p* ≤ 0.05, ** *p* ≤ 0.01, *** *p* ≤ 0.001. Statistical comparisons (**A**,**C**–**E**) performed using Student’s *t*-test; (**B**) comparison of sigmoid dose–response models.

As chronic exposure of pancreatic cancer cells to hypercapnia (10% CO_2_) was previously reported by us to elicit an aggressive phenotype associated with increased growth and platinum resistance [5], we first began by validating this phenotype in pancreatic cancer cells. Pancreatic cell lines exposed to chronic hypercapnia displayed a marked increase in cell growth in a confluency-independent matter (Figure 1A). Oxaliplatin cytotoxicity was also assessed, with hypercapnic conditions showing a 2–3 fold increase in the IC50 compared to normocapnic culturing conditions (Figure 1B). As chronic hypercapnia has been shown to induce expression of HIF1α in pulmonary and cerebral tissues [22,23], we measured its protein expression in PDAC cells and also found it to be increased in hypercapnic conditions (Figure 1C).

We previously showed that PDAC tumors with increased expression of hypercapnic gene signatures were associated with higher lymph node burden and worse survival outcomes [6]. Here, we examined the impact of chronic hypercapnia on the migration and invasion of PDAC cells. Scratch migration assay showed increased cell migration across three different pancreatic cell lines (MiaPaCa2, Panc1, AsPC-1), as seen in Figure 1D. We next assessed the effect of chronic hypercapnia on cell invasion. Drawing upon the findings of Obata et al. [3], who reported on increased invasion of colon cancer cells exposed to hypercapnia, we performed Matrigel trans-well invasion assays and found increased cell invasion in MiaPaCa2 PDAC cells exposed to 10% CO_2_ hypercapnia (Figure 1E).

### 2.2. Gene Expression Analysis Shows SIAH3 as the Most Differentially Regulated Transcript in Chronic Hypercapnia

**Figure 2:**
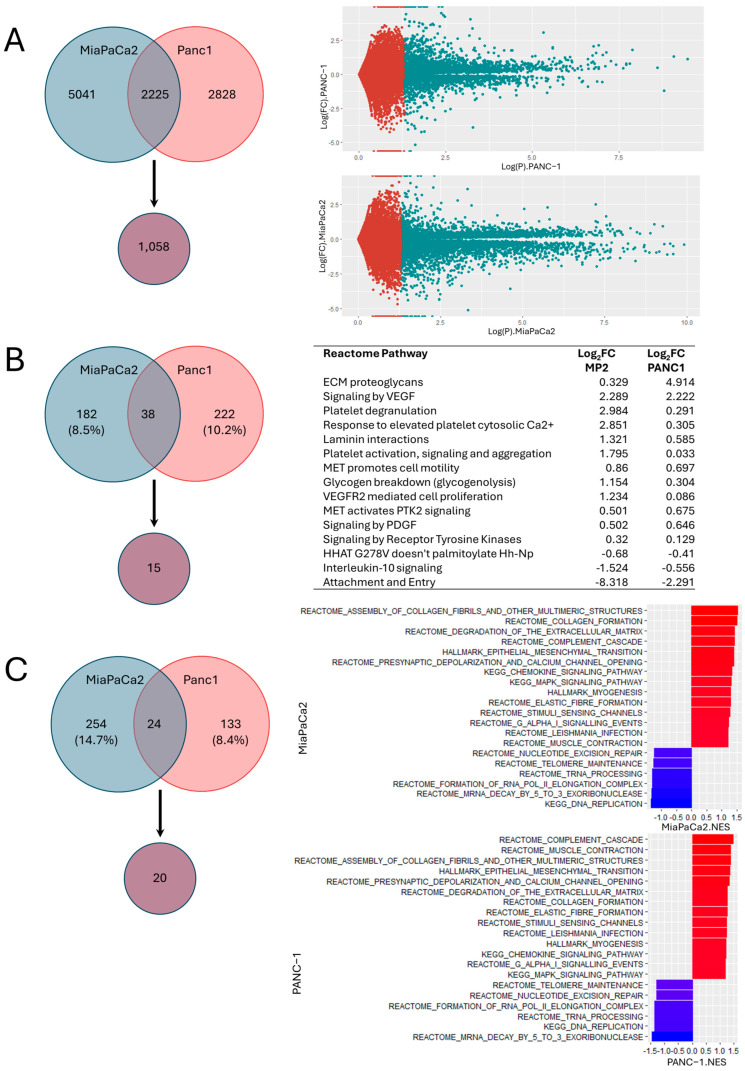
Gene expression analysis of PDAC cell lines (MiaPaCa2, Panc1) identifies distinct changes in extracellular matrix regulation, epithelial-to-mesenchymal transition, and proinflammatory signaling. PDAC cell lines were chronically cultured in normocapnia conditions (5% CO_2_, normocapnia [control]) and compared to culturing in hypercapnia conditions (10% CO_2_). (**A**) Differential gene expression cross-validated between both cell lines for significance (Nominal *p*-value ≤ 0.05) and concordant response, identified 1058 differentially expressed genes; the right panel shows volcano plots for each cell line (significant targets are in teal). (**B**) Reactome pathway analysis, cross-validated between both cell lines for significance (Adjusted *p* ≤ 0.05), and concordant response identified 15 Reactome pathways (listed in the right panel table). (**C**) Gene set enrichment analysis (GSEA), cross-validated between both cell lines for significance (Nominal *p*-value ≤ 0.05) and concordant response identified 20 signatures. The normalized enrichment score of the cross-validated pathways is shown on the right for each cell line.

The effects of chronic hypercapnia (5% CO_2_ vs. 10% CO_2_) on gene expression were assessed via whole exome RNA sequencing (RNA-Seq). The analysis was performed across two PDAC cancer cell lines (MiaPaCa2 and Panc1) in order to validate significant, concordantly affected targets. A total of 5041 and 2828 differentially expressed genes were noted in the hypercapnic MiaPaCa2 and Panc1 cells, respectively. Comparison of both sets of differentially expressed genes yielded 1058 genes that were significantly and similarly impacted by chronic hypercapnia in both PDAC cell lines (Figure 2A). The three topmost differentially expressed transcripts across both PDAC cell lines were *SIAH3*, *C3orf80*, and *EML5* (Table 1, Supplementary Tables S1 and S2).

As SIAH3 was the topmost differentially expressed transcript and the SIAH protein family members (SIAH1, SIAH2) are known downstream targets and regulators of the RAS-MAPK pathway [24,25,26,27,28], we considered this potential target as highly interesting.

### 2.3. Molecular Pathway Analysis Shows Activation of EMT and TME Regulatory Pathways

A Reactome pathway analysis (www.reactome.org; accessed on 26 November 2024) was performed on the RNA-seq data. A total of 220 (8.5%) and 260 (10.3%) differentially expressed Reactome pathways were noted in the hypercapnic MiaPaCa2 and Panc1 cells, respectively. Of these pathways, 15 pathways were validated across both cell lines and had concordant changes upon chronic exposure to hypercapnia (Figure 2B). The top three impacted Reactome pathways included up-regulation of the “ECM proteoglycans” and “Signaling by VEGF” pathways and down-regulation of the SARS-CoV-2 associated “Attachment and Entry” pathway.

Additional GSEA of KEGG, Reactome, and Hallmark pathways was employed, identifying 278 (14.7%) and 157 (8.4%) differentially expressed gene sets in the hypercapnic MiaPaCa2 and Panc1 cells, respectively. Of these, 20 gene sets were found to be significant and concordantly impacted across both cell lines (Figure 2C). Importantly, this set of impacted pathways includes increased expression of ECM, collagen, and elastic fiber regulatory gene sets along with the hallmark epithelial-to-mesenchymal (EMT) transition gene set, which includes SNAI2, FOXC2, FN1, VIM, LAMA2, LAMA3, LAMAC2, and ACTA2 (Supplementary Figure S1). This further supports the phenotypic changes to cellular migration and invasion we have witnessed in PDAC cells exposed to hypercapnia and poses a potential mechanism for the aggressive disease progression witnessed in patients with hypercapnic tissue signatures [6].

### 2.4. Chronic Hypercapnia Downregulates SIAH3 Gene mRNA and Protein Expression

**Figure 3:**
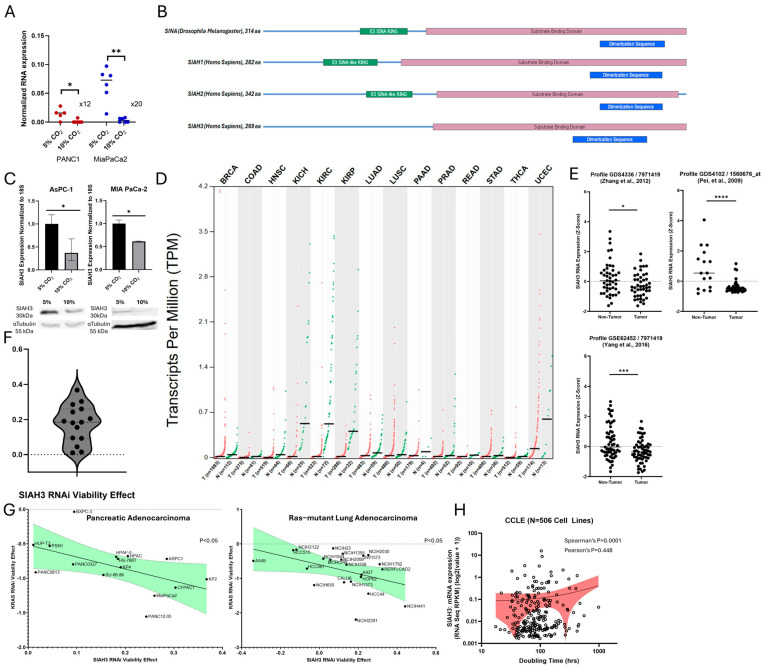
SIAH3 is a target of chronic hypercapnia that is down-regulated in cancer and is negatively associated with cancer cell growth. (**A**) Normalized counts of SIAH3 expression from whole exome RNA-sequencing comparing expression in chronic hypercapnia and normocapnia conditions (10% CO_2_ vs. 5% CO_2_) in two PDAC cell lines (MiaPaCa2 and Panc1). (**B**) cDNA sequences of SINA (Drosophila Melanogaster), SIAH1, SIAH2, and SIAH3 showing conserved sequence elements. (**C**) Top panel, RT-qPCR expression of SIAH3 in two pancreatic cancer cell lines (AsPC-1 and MiaPaCa2) chronically cultured in hypercapnia and normocapnia conditions (10% CO_2_ vs. 5% CO_2_) along with concordant protein expression (bottom panel). (**D**) SIAH3 expression in normal and tumor tissues (TCGA). (**E**) SIAH3 in normal and tumor pancreatic tissues in three GEO datasets [32,33,34]. (**F**) DepMap RNAi analysis (Achilles + DRIVE + Marcotte) of the viability effect of SIAH3 RNAi across 15 pancreatic cancer cell lines. (**G**) DepMap RNAi analysis of viability response correlation between SIAH3 and KRAS in 15 pancreatic cell lines (left) and 22 RAS-driven lung adenocarcinoma cell lines (right). (**H**) CCLE analysis correlation between SIAH3 RNA expression and doubling time in cancer cell lines (N = 506). * *p* ≤ 0.05, ** *p* ≤ 0.01, *** *p* ≤ 0.001, **** *p* ≤ 0.0001. Statistical comparisons (**A**,**C**,**E**) are performed using Student’s *t*-test and correlative analysis with Pearson correlation tests (**G**) and the Spearman correlation test for non-linear correlation (**H**).

Next, we attempted to use the RNA-seq data to identify possible downstream targets of hypercapnia signaling. In order to identify the most impacted genetic targets, we assessed the combined fold change in our differentially expressed gene across both of the tested pancreatic cancer cell lines (FC_MiaPaCa2_ × FC_Panc1_). We chose to examine the topmost impacted genetic target, specifically the seven-in-absentia (SINA) homolog family member 3 (SIAH3), which was found to be greatly down-regulated in chronic hypercapnia (Table 1, Figure 3A). SIAH3 is a catalytically inactive, lesser-known member of the SIAH family of ubiquitin E3 ligases (Figure 3B). While both SIAH1 and SIAH2 are known pro-tumorigenic factors, hypoxia-sensors, and downstream effectors of the Ras-Raf-Mek pathway, SIAH3 has been considered to have no distinct E3 ligase activity and has been mostly investigated regarding its role in the regulation of mitophagy [29,30,31]. Some studies have even pointed to SIAH3 as a possible competitive inhibitor of SIAH1 function by dimerizing with SIAH1 and sequestering it to the mitochondria [29]. As pancreatic cancer is primarily driven by the overactivation of the RAS pathway, and the SIAH family has a known role in the hypoxia signaling cascade, we found these changes in SIAH3 expression to be especially interesting.

We first validated our RNA-Seq results with RT-qPCR and western blots, showing a significant decrease in RNA and protein expression of SIAH3 in cells exposed to chronic hypercapnia (Figure 3C). Data from the Tumor Cancer Genome Atlas (TCGA) cohort showed SIAH3 to be down-regulated in multiple cancers, including pancreatic adenocarcinoma (PAAD), compared to normal non-cancerous tissues (Figure 3D). This was further corroborated by examining pancreatic RNA expression data from patients’ tumors and normal tissues from two available GEO sets, published by Zhang et al. [32] (*p* < 0.05), Pei et al. [33] (*p* < 0.0001), and Yang et al. [34] (*p* < 0.001) (Figure 3E). Additional analysis of the DepMap portal revealed that siRNA knockdown of SIAH3 resulted in a net increase in cell proliferation across all 15 pancreatic cancer cell lines (Figure 3F). Furthermore, the improved viability in PDAC cell lines was inversely correlated to the cell lines’ response to KRAS inhibition (ρ = −0.532, *p* = 0.041), as seen in Figure 3G (left panel). A similar relationship is seen in RAS-mutated lung cancer cell lines (Figure 3G, right panel, *p* = 0.04). This inverse relationship between KRAS knockdown and SIAH3 knockdown suggests a possible shared pathway, with SIAH3 acting as a negative-feedback or a counter-regulatory mechanism against the downstream effects of the KRAS-MEK-MAPK pathway. Tang et al. proposed that the SIAH proteins (SIAH1/SIAH2) can serve, essentially, as gatekeepers for the KRAS-MEK-MAPK pathway [35]. Additionally, SIAH3, which lacks the otherwise conserved RING domain, has been reported to trap SIAH1 via dimerization and to localize it to the mitochondrion, thereby preventing the cytoplasmic ubiquitination activity of SIAH1 and regulating mitophagy [29], making SIAH3 into a potential negative regulator of the pathway. Since the RAS-MEK-MAPK pathway is an important regulator of cell proliferation in cancer cells [36,37], we analyzed data from the Cancer Cell Line Encyclopedia (CCLE) for correlation between SIAH3 expression and cellular growth rates in 506 cancer cell lines (Figure 3H). A positive correlation was noted between SIAH3 expression and doubling time (Spearman’s ρ = 0.170, *p* = 0.0001), but not in a linear fashion (Pearson’s ρ, *p* = 0.448). Control analyses for known cell cycle up-regulators (KI-67) and down-regulator (CDKN1A) are available in Supplementary Figure S2A,B. This suggests that rapidly replicating cancer cell lines exhibit low expression levels of SIAH3.

### 2.5. SIAH3 Expression Is a Positive Prognostic Marker of Pancreatic Adenocarcinoma Survival

**Figure 4:**
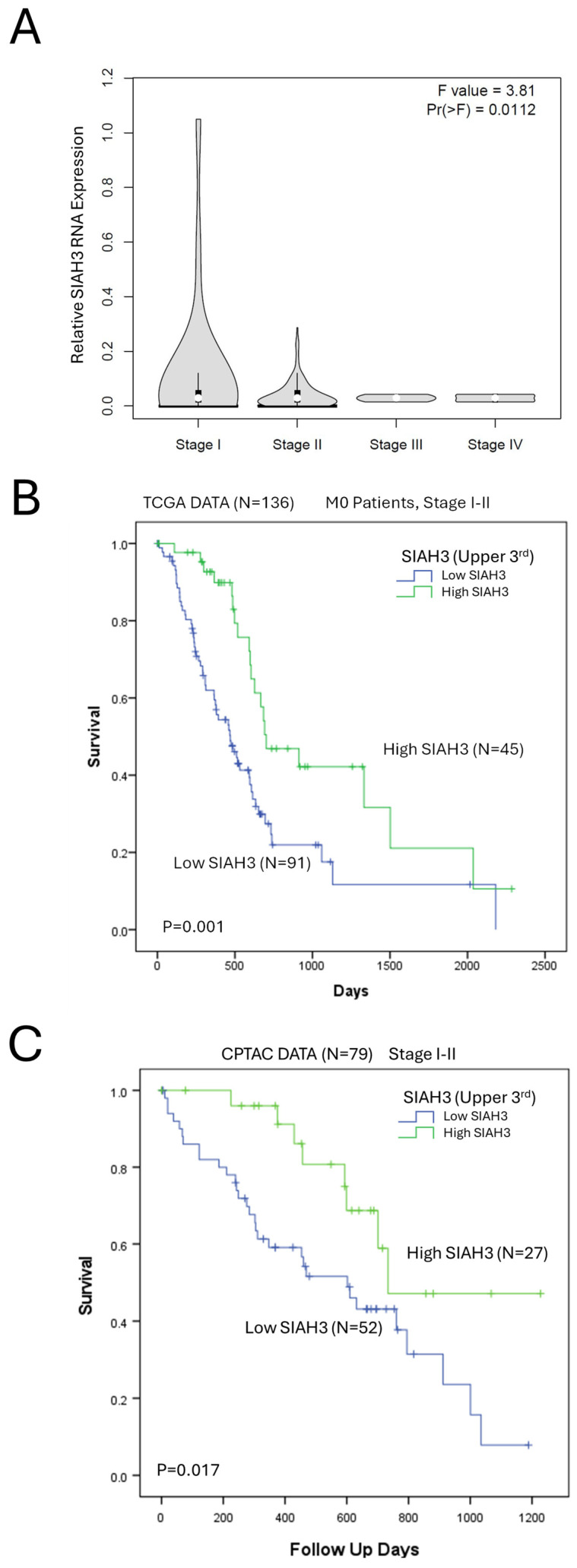
SIAH3 expression is a favorable prognostic factor in PDAC. (**A**) SIAH3 RNA expression decreases over pancreatic cancer stages (TCGA-PAAD, N = 179), compared by one-way ANOVA (*p* = 0.011) and Pearson’s correlation test (*p* = 0.014). (**B**) Kaplan–Meier survival analysis comparing survival in non-metastatic, stage I-II patients with PDAC tumors presenting high (upper tertile) vs. low (1st and 2nd tertiles) of SIAH3 RNA expression (TCGA-PAAD, N = 134), compared using the log-rank test. (**C**) Kaplan–Meier survival analysis comparing survival in non-metastatic, stage I–II patients with PDAC tumors presenting high (upper tertile) vs. low (1st and 2nd tertiles) of SIAH3 RNA expression (CPTAC, N = 79), compared using the log-rank test.

TCGA data of the entire PAAD (pancreatic adenocarcinoma) cohort showed SIAH3 expression to be decreased in advanced cancer stages (*p* < 0.01, Figure 4A), suggesting a possible correlation with disease progression. We, therefore, assessed SIAH3 expression as a clinical prognostic marker in PDAC across two clinical cohorts (TCGA and CPTAC). Kaplan–Meier analyses show that high expression of SIAH3 (upper tertile) is associated with significantly improved overall survival in non-metastatic PDAC patients in both the TCGA and the CPTAC pancreatic adenocarcinoma cohorts (Figure 4B,C). Cox hazard analyses of the TCGA and CPTAC pancreatic cancer cohorts identified SIAH3 as a positive prognostic marker for overall survival (HR = 0.4 and HR = 0.36, respectively), as shown in Table 2. Further Cox analysis to include KRAS subtype status and tumor grade was performed on the TCGA cohort after the exclusion of low-occurring KRAS subtypes (G12A, G12C, Q61R). The resulting model suggested that high SIAH3 expression corresponded with improved survival (HR 0.36, *p* = 0.0007. Supplementary Table S3).

## 3. Discussion

The vast existing research regarding hypoxia has established that the respiratory tumor microenvironment plays a significant role in cancer progression through multiple direct and indirect signaling pathways. In several pulmonary disorders and in the context of physiologic tissue perfusion, hypoxia is frequently accompanied by hypercapnia [38,39,40,41]. Surprisingly, considerably less data are available regarding the impact of carbon dioxide (and hypercapnia) on cancer progression and on the TME. In fact, NIH PubMed queries comparing the net yield of “hypoxia” and “cancer” vs. “hypercapnia” or “hypercarbia” and “cancer” reveals a ratio of over 80:1 in favor of hypoxia-related studies. In recent years, with the establishment of hypoxia signaling as a possible therapeutic target, this ratio has increased to almost 150:1.

The effects of hypercapnia appear to be tissue and cell-type dependent: Tsuji et al. [10] noted that hypercapnia induces an increase in the proliferation of HUVEC cells without any significant impact on cell migration. However, Vohwinkel et al. [13] described hypercapnic conditions to inhibit the proliferation of fibroblast (N12) and lung epithelial cells (A549), and Dada et al. [19] found hypercapnia to inhibit the proliferation of lung alveolar type 2 (AT2) cells through stromal regulation of the WNT-β catenin pathway. Further clinical support for the findings of Vohwinkel et al. and Dada et al. can be found in the report of Bharat et al. [42], which shows that pleural hypercapnia slowed down the repair of alveolo-pleural fistulas. Cell migration also appears to be impacted by hypercapnia, with lung epithelial cells showing decreased motility under hypercapnic conditions through an NF-kappaB mediated inhibition [43].

Contrary to these observations in non-neoplastic cells, hypercapnia appears to have significant pro-tumorigenic effects in cancer cells. Obata et al. [3] and Zhao et al. [44] noted that exposure to high levels of carbon dioxide results in increased migration of colon cancer cells in vitro, which was mediated through the upregulation of MMP9. Hypercapnia-induced cell migration was also noted in GIST cells in vitro [45] and in a co-culturing model of neuroblastoma cells with peritoneal mesothelial cells [46].

We previously reported on hypercapnia as an inducer of increased cell proliferation and oxaliplatin drug resistance in PDAC cells [5]. In the current study, we found that pancreatic cancer cells cultured in-vitro, in chronic hypercapnia, respond with increased cell migration and invasion, which are cancer hallmarks of an aggressive phenotype.

Comparison of hypercapnia to hypoxia shows that the effects are either agonistic or synergistic in a context-dependent fashion: In the current study, we note an increased expression of HIF1α in cells exposed to prolonged hypercapnia. This finding is interesting as in some non-cancer studies, hypercapnia was shown to be associated with suppression of the HIF1α axis [47]. However, increased levels of HIF1α in hypercapnia are concordant with other previous physiological studies and even in non-hypoxic models [22]. This increased expression of HIF1α may account for a part of the aggressive phenotype we have seen in pancreatic cancer cells in-vitro and may shed additional light on the experimental observations of increased cell invasion and marked HIF1α overexpression after exposure to CO_2_ pneumoperitoneum (which mimics combined hypoxia and hypercapnia) [44,48,49]. Next, our transcriptomic analyses indicated that chronic hypercapnia results in the activation of pathways associated with TME remodeling, MET-associated cell migration, and activation of hypoxia-associated growth factor pathways (VEGF and PDGRF). These changes to the ECM are in line with some of the pulmonary changes found in mouse pups exposed to hypercapnia, as described by Li et al. [17] and Ryu et al. [18].

When considering the studies showing that hypercapnia, like hypoxia, modulates WNT signaling in the lungs [19] and induces upregulated HIF1α signaling and VEGF expression [7], a shared pattern begins to emerge. On the other hand, while hypoxia is associated with promoting the proliferation of lung fibroblasts and pneumocytes, hypercapnia appears to be associated with inhibition of cell growth in those tissues [13,19]. Hypoxia is generally associated with decreased proliferation in other tissues [50,51]. However, mild hypoxia has been shown to promote proliferation and cell stemness in some cancer cells and stem cells [52,53,54,55]. Our results indicate that chronic hypercapnia results in increased proliferation in PDAC cell lines. Similarly, hypercapnia has been shown to induce immune suppression, while hypoxia has been shown to have a complex net effect combining activation of some immune elements [56,57] while suppressing others [58,59]. Our GSEA analyses suggested that chronic hypercapnia induces increased expression of the Reactome complement cascade pathway and the KEGG chemokine signaling pathway. These findings support the notion of hypercapnia signaling involving a distinct and separate pathway from that of HIF1α. Overall, these observations possibly suggest that both triggers, hypoxia and hypercapnia, act as agonists in certain settings and as opposing regulators in other settings.

These observational characterizations of cellular response to hypercapnia hold direct relevance to the clinical environment of solid tumors. Hypercapnia has been reported in the tumor microenvironment of solid cancers [1,2,60] and is postulated to occur as a result of endogenous tumor activity in the setting of decreased elimination of metabolic waste products [60,61]. While there have been no interventional studies that targeted the hypercapnic microenvironment, some indirect evidence may be found in retrospective analyses: Obstructive respiratory diseases have been linked to poorer oncological outcomes in PDAC [5,62]. Additionally, hypercapnic gene signatures in PDAC tissues have been shown to be associated with earlier recurrence and a more aggressive disease course [6].

As an initial exploration into hypercapnia signaling in PDAC, we assessed the inactive E3 ligase member of the SINA homolog family, SIAH3, which was shown to be greatly downregulated in PDAC cells exposed to chronic hypercapnia (Log_2_[Fold-change]: 3.2–4.3, MIA-PaCa-2 *p* = 5.1 × 10^−6^, PANC-1 *p* = 0.0186). SIAH3 appears to be an attractive target for further investigation as its active homologs, SIAH1 and SIAH2, are known to be downstream effectors of the KRAS pathway [63] and have been shown to be associated with cancer progression and poor prognosis in multiple cancers [27,64,65,66,67,68]. SIAH1 and SIAH2 are also regulators of the hypoxia signaling pathway, controlling prolyl hydroxylase stability and HIF1α expression [25,28,67].

DepMap data suggest that inhibition of SIAH3 results in proliferation in PDAC cells and in RAS-driven lung cancer cells (Figure 3G), and TCGA clinical samples show a progressive decrease in SIAH3 expression in advanced PDAC stages (Figure 4A). However, we have not validated these results in our current study, and the exact association between the RAS pathway and SIAH3 remains to be understood. Clinically, our analyses show that high SIAH3 serves as an independent positive prognostic marker of overall survival in PDAC patients in two separate clinical datasets (TCGA and CPTAC). It is still unclear whether SIAH3 holds any mechanistic and clinical impact on cancer progression or whether it serves only as an associated prognostic factor and further mechanistic studies should be undertaken to elucidate its role.

Multiple molecular prognostic factors have been identified in PDAC. Of these, mutations in KRAS, P53, and SMAD4 are considered key risk factors [69,70,71]. Secretory factors such as mucin and others were also shown to correspond with PDAC survival [72,73,74]. Our data show and validate SIAH3 as a fairly substantial risk factor in our cohorts of early-stage PDAC. While we were unable to access treatment records in order to include those in our regression model, it would be interesting to see them in larger studies.

Several limitations of our study merit further discussion: First, the emerging data suggest that hypercapnia effects are tissue-specific and, therefore, may not be generalizable to all cancer models: for example, hypercapnia effects in pancreatic cancer may differ than in lung cancer or breast cancer. Additionally, our work was based on in vitro cell line experiments without modeling the stromal microenvironment and without supplementary in vivo assessments, which are planned as our next investigational steps. The transcriptomic-based pathway analyses, while in line with previously published hypercapnia observations in other tissues [17,18], were not further validated with protein expression in this study. Our list of hypercapnia-affected targets, identified via RNA-seq, contained a large number of potential targets, of which we only chose to explore the topmost differentially expressed target. This limited investigation, performed as a proof-of-concept for the use of chronic hypercapnia to screen for cancer-related targets, validated SIAH3 as a hypercapnia-effected target and observed an association with clinical outcomes. However, the mechanism of action of SIAH3 in PDAC was not explored, nor was the causality. These experiments are planned to be performed in our future work. Clinically, we need to note that both of our cohorts were composed of non-metastatic, early-stage pancreatic cancer patients. This, therefore, also limits the generalizability of our results. We hope that with the inclusion of bigger, more detailed patient cohorts, subsequent survival analyses will account for all disease stages, treatment history, and additional molecular biomarkers.

In summary, our data strongly suggest that chronic exposure to hypercapnia (10% CO_2_ vs. 5% CO_2_) triggers an aggressive phenotype in PDAC cells, which includes increased proliferation, oxaliplatin resistance, increased invasion, and cell migration. Concordantly, expression pathway analyses point towards the activation of extracellular matrix remodeling pathways, cell motility pathways, epithelial-to-mesenchymal transition, and VEGF and PDGF-associated pathways. Our preliminary analysis of the top impacted genetic target of hypercapnia, the E3-ligase inactive member of the SIAH family, SIAH3, revealed it to be associated with cell proliferation and inversely proportional in its effect on the function of KRAS. Clinically, high SIAH3 expression appears to be a strong positive prognostic factor for overall survival in non-metastatic PDAC. To better investigate the impact of hypercapnia on the TME, our future and ongoing studies will assess the in vivo effects of hypercapnia in xenograft and allograft mouse tumor models.

## 4. Materials and Methods

### 4.1. Cell Culture

Cell lines were obtained from the American Type Culture Collection (Manassas, VA, USA). MIA-PaCa-2, PANC-1, and AsPC-1 cells were grown in standard Dulbecco’s modified Eagle’s medium-based media (DMEM), supplemented with 10% fetal bovine serum, 1% L-glutamine, and 1% penicillin–streptomycin (Thermo Fisher Scientific, Waltham, MA, USA) and 5 mg/L Plasmocin (Invivogen, San Diego, CA, USA). All cells were regularly tested for mycoplasma using the LookOut mycoplasma PCR detection kit (Millipore-Sigma, St. Louis, MO, USA). Cells were passaged when between 50% and 75% confluent and discarded at a maximum passage number of 18. All cells were cultured in standard conditions (humidified incubators at 5% CO_2_ and 37 °C) unless otherwise specified.

### 4.2. Cell Culturing with CO_2_ Exposure

All cells were grown in humidified CO_2_ incubators at 37 °C. A CO_2_ concentration of 10% was used to mimic hypercapnic conditions. All media used for culturing hypercapnic cells was exposed to 10% CO_2_ for at least 4 hours prior to use to decrease changes in dissolved CO_2_. Cells from the normocapnia (control) group were grown in 5% CO_2_. DMEM media is bicarbonate buffered and optimized for cell culturing in 5–10% CO_2_ to result in physiological pH levels. However, we also assessed the growth media pH with a pH-meter (Corning pH-Meter 440, Corning, Tewksbury, MA, USA) and pH indicators (Hydrion PH 5.5–8.0 and Hydrion PH 6.0–8.0, Micro-Essential Laboratory, Brooklyn, NY, USA) to validate physiologic pH culturing conditions (Supplementary Figure S3). All chronic hypercapnia experiments included pre-culturing the cells in 10% CO_2_ for 10–14 days, in accordance with previous reports indicating a shift in the gene expression profile between acute and chronic hypercapnia [23].

### 4.3. Colony Formation Assays

MIA-PaCa-2 (MiaPaCa2), PANC-1 (Panc1), and AsPC-1 cells chronically grown in either 5% or 10% CO_2_ were plated in 6-well dishes. Cells were plated at 1000 cells per well 24 h prior to drug exposure. Cells were then dosed with varying concentrations of oxaliplatin (HY-17371, MedChemExpress, Monmouth Junction, NJ, USA). For PANC-1 and AsPC-1 cells, media was changed and oxaliplatin re-administered on day 7, and the experiment was terminated on day 14. For MIA-PaCa-2 cells, media was changed and oxaliplatin re-administered on day 5, and the experiment was terminated on day 10. For the proliferation study, MIA-PaCa-2 and PANC-1 cells were plated at multiple cell densities (100, 400, 600, and 1000 cells per well) and maintained in the same manner described above without exposure to oxaliplatin. At termination, cells were washed in DPBS, fixed with 80% methanol, stained with 0.5% crystal violet in 20% methanol, washed in deionized water, and air dried. For each well, the colony surface area was quantified with ImageJ (version 1.54g, National Institute of Health, Bethesda, MD, USA). All experiments were performed in triplicates.

### 4.4. Cell Migration (Scratch Wound) Assay

MIA-PaCa-2 and AsPC-1 cells that were chronically grown in either 5% or 10% CO_2_ were plated at 3 × 10^6^ cells in 6 cm dishes 24 h prior to scratch administration. Two perpendicular wounds were made, which bisected the center of the plate. Images of the intersecting scratches were captured every 24 h over 4 days. Analysis was performed by measuring the distance between scratch edges at 6 separate points along the scratch and averaging the results. Image capture and analysis were performed for each day. Quantification of the wound area was performed in ImageJ (version 1.54g, NIH, Bethesda, MD, USA). All experiments were performed in triplicates.

### 4.5. Cell Transwell Invasion Assay

The assay was performed using Matrigel Boyden Chambers as per the manufacturer’s instructions. Briefly, cells that were chronically grown in normocapnic or hypercapnic conditions were serum-starved overnight. A total of 5 × 10^4^ cells in serum-free media were placed in the top well of Matrigel invasion chambers (BD Biosciences, Chicago, IL, USA), with 20% FBS media (chemo-attractant) in the lower chamber. Cells were allowed to invade/migrate through the membrane for 24 h at 37 °C. After 24 h, cells on the upper surface of the membrane were removed with cotton swabs, membranes were cut, and cells on the undersurface of the membranes were fixed in 100% methanol, stained with crystal violet, and all cells were counted manually.

### 4.6. RT-qPCR and RNA Quantification

MIA-PaCa-2 and AsPC-1 cells chronically grown in either 5% or 10% CO_2_ were plated at 3 × 10^6^ in 15 cm dishes, and RNA was extracted after 72 h (RNeasy Mini Kit, Qiagen, Hilden, Germany). In order to overcome low transcript levels of the SIAH3 target, preparation of cDNA was performed by replacing the random primer component of a commercially available reverse transcription kit with sequence-specific primers specific to SIAH3 and 18S and using 2 μg of total RNA with the Applied Biosystems High-Capacity cDNA Reverse Transcriptase kit (Life Technologies Corp., Carlsbad, CA, USA). Transcripts were quantified using SYBRTM Select Master Mix and QuantStudio 3 Real-Time PCR (Applied Biosystems, Waltham, MA, USA). Quantitative PCR (RT-qPCR) was performed as previously described [75,76], and relative quantification was performed using the 2-ΔΔCt method.

Specific forward and reverse primers for RT-qPCR analysis were as follows:

18S: 5′-GCTTAATTTGACTCAACACGGGA-3′ and 5′-AGCTATCAATCTGTCAATCCTGTC-3′. SIAH3: 5′-ACTGCTTCACCTATCGCCTG-3′ and 5′-TAATCACCGAGTCCACGCAC-3′.

### 4.7. Whole Exome Sequencing and Gene Expression Analysis

Gene expression analysis was assessed through whole-exome RNA sequencing in both MIA-PaCa-2 and PANC1 PDAC cells. Each experimental arm was repeated in six biological replicates. Cultured cells, continuously propagated in their respective atmospheres (5% CO_2_ vs. 10% CO_2_), were plated at 3 × 10^6^ cells/15 cm plate, cultured for 48 h, and collected for total RNA extraction (RNeasy mini kit, Qiagen, Germantown, MD, USA). Whole-exome RNA sequencing and count normalization were performed by Novogene (Novogene Corporation Inc., Durham, NC, USA) utilizing the Illumina Platform PE150. Differential expression analysis of two atmospheric conditions was performed using the DESeq2 R package (1.36.0). As an alternative approach to false discovery rate (FDR) correction, expression data from the two separate PDAC cell lines were cross-referenced for nominal *p*-value ≤ 0.05 and for concordant fold-change between the conditions (i.e., up-regulation vs. down-regulation), thereby using the results of the RNA-seq in the two individual cell lines to, more stringently, cross-validate each other. Genetic targets found to be significant in both cell lines and concordant in their response were considered cross-validated targets of the RNA-Seq (Supplementary Tables S1 and S2).

### 4.8. Reactome Pathway Analysis

A Reactome pathway analysis (www.reactome.org, accessed on 26 November 2024) was performed on the RNA-seq data from two cell lines (MIA-PaCa-2 and PANC1). Briefly, a competitive gene-set test accounting for inter-gene correlation (“camera” function, “limma” package) was performed on the normalized FPKM RNA counts, allowing for a proportion of up to 0.5 (50%) missing values per group. A ‘TMM’ discrete normalization function was used on the data.

Data from two separate PDAC cell lines were cross-referenced for FDR-adjusted *p*-value ≤ 0.05 and concordant fold-change between the conditions (i.e., up-regulation vs. down-regulation). Reactome pathway signatures that were found to be significant in both cell lines and concordant in their response were considered cross-validated.

### 4.9. Gene Set Enrichment Analysis (GSEA)

GSEA (GSEA, version 4.0, Broad Institute, Cambridge, MA, USA) was performed on RNA-Seq data comparing gene expression of PDAC cells (MIA-PaCa-2 and PANC1) cultured in chronic hypercapnia (10% CO_2_) vs. normocapnia (5% CO_2_). Gene sets used were obtained from the Molecular Signatures Database (MSigDB, v 6.0; https://www.gsea-msigdb.org, accessed on 30 July 2024), including cancer hallmarks, Reactome pathways, and KEGG signatures. Data from two separate PDAC cell lines were cross-referenced for nominal *p*-value ≤ 0.05 and concordant fold-change between the conditions (i.e., up-regulation vs. down-regulation). GSEA signatures that were found to be significant in both cell lines and concordant in their response were considered cross-validated signatures.

### 4.10. Immunoblot Analysis

Cells were lysed in ice-cold RIPA buffer (Santa Cruz Biotechnology, Dallas, TX, USA) supplemented with PMSF, protease inhibitor cocktail, and phosphatase inhibitor (Thermo Scientific, Waltham, MA, USA). Protein concentration was determined by the Pierce BCA Assay (Thermo Scientific, Waltham, MA, USA), and samples of equal concentration were prepared in 5× Laemmli buffer (Thermo Scientific Chemicals, Waltham, MA, USA). Samples were resolved by SDS-PAGE gel electrophoresis and transferred to a PVDF membrane. Membranes were then blocked with a commercially available product (Intercept Blocking Buffer, LI-COR Biosciences, Lincoln, NE, USA). The SIAH3 primary antibody (NBP2-83524, Novus Biologicals, Minneapolis, MN, USA) was diluted 1:1000 in blocking buffer, the α-Tubulin primary antibody (32-2500, Invitrogen, Waltham, MA, USA) was diluted 1:2500 in blocking buffer, and the HIF1α primary antibody (PA1-16601, ThermoFisher Scientific, Waltham, MA, USA) was diluted 1:1000 in blocking buffer. Appropriate fluorescent-conjugated secondary antibodies (Li-COR Biosciences, Lincoln, NE, USA) were diluted 1:500, and images were captured by ChemiDoc MP Imaging System (Bio-Rad Laboratories, Hercules, CA, USA).

### 4.11. Normal and Tumor Tissue Expression Data

The GEPIA2 portal (http://gepia2.cancer-pku.cn/, accessed on 26 November 2024) was used to compare SIAH3 expression levels in tumor and normal tissues in the TCGA datasets and across multiple cancer stages in the PAAD set. Two gene expression omnibus (GEO) datasets were also used to compare SIAH3 expression levels between normal and tumor pancreatic tissues (GSE16515, GSE28735) [32,33,34] with the Zhang et al. and Yang et al. sets compared in a pairwise fashion as per its original design and the Pei et al. compared in an unpaired fashion.

### 4.12. RNAi Viability Effect Analysis

The effect of RNA inhibition on SIAH3 was assessed via the RNAi viability screen data available on the DepMap portal (www.depmap.org, accessed on 26 April 2024). Briefly, RNAi data from three sources (Achilles project [77], DRIVE project [78], and Marcotte et al. [79]) were analyzed using the DEMETER2 Bayesian analytical framework [80]. Pancreatic adenocarcinoma cell lines and lung adenocarcinoma cell lines were reviewed, and SIAH3 RNAi and KRAS RNAi data were recorded.

### 4.13. CCLE Analysis of Cell Line Growth Rates

Data regarding cancer cell line growth rate (i.e., doubling times) and RNA-seq-based mRNA expression values (RPKM) were available from the Cancer Cell Line Encyclopedia (CCLE) [81]. RNA expression values of SIAH3 were compared to cell line growth rates across 506 available cancer cell lines. The correlation was analyzed using linear regression analysis and with Spearman’s correlation test.

### 4.14. Clinical Data Collection

Clinical and genomic data were retrieved from the online Tumor Cancer Genome Atlas (TCGA) pancreatic adenocarcinoma dataset (PAAD) and the Clinical Proteomic Tumor Analysis Consortium (CPTAC) pancreatic cancer dataset. Patients with pancreatic tumors other than pancreatic adenocarcinoma were excluded. In the TCGA and the CPTAC cohorts, patients with undetermined staging, stage III, or stage IV, were excluded from analysis.

### 4.15. Statistical and Survival Analysis

Categorical data were compared using the chi-square test. Continuous variables were assessed for normality of distribution with the Kolmogorov–Smirnov test. Normally distributed continuous variables were compared using a Student’s *t*-test. Numeric variables with a nonparametric distribution pattern were compared using a Mann–Whitney test. Kaplan–Meier survival analyses (with log-rank comparisons) were used to compare different survival risk factors for overall survival and disease-specific survival. Cox-hazard survival analyses were performed. Iterative exclusion of variables from the analysis was performed based on the Akaike information criterion and *p*-value until the optimal model was achieved. *p* values equal to or lesser than 0.05 were considered statistically significant. Statistical analyses were performed using the Statistical Package for Social Sciences (IBM SPSS, version 20, SPSS Inc., Chicago, IL, USA) and GraphPad Prism (Version 10, Boston, MA, USA).

## Figures and Tables

**Table 1:** Topmost differentially expressed transcripts across MiaPaCa2 and Panc1 PDAC cell lines under chronic hypercapnia exposure (10% CO_2_) compared to normocapnia (5% CO_2_).

Transcript	Log_2_ (Fold Change) MiaPaCa2	Log_2_ (Fold Change) Panc1
SIAH3/ENSG00000215475	−4.283	−3.175
EML5/ENSG00000165521	−1.892	−2.393
C3ORF80/ENSG00000180044	−2.179	−2.328

**Table 2:** Cox survival regression assessing the impact of increased tumor expression SIAH3 (upper tertile) across two pancreatic cancer cohorts (TCGA, CPTAC) in patients with early-stage (TNM stage I–II) pancreatic cancer. R—completeness of resection (e.g., R0—complete resection, R1—microscopically positive resection margin, R2—gross tumor macroscopic tumor remaining). HR—hazard ratio.

TCGA (N = 134)		
Covariate	HR	Sig.
T	1.26 (0.71–2.26)	0.431
N	0 (0–0)	0.047
N1	1.47 (0.81–2.65)	0.205
NX	12.8 (1.54–106.28)	0.018
High SIAH3	0.4 (0.24–0.67)	0.001
CPTAC (N = 79)		
Covariate	HR	Sig.
T stage	1.821 (0.98–3.36)	0.058
N stage		0.178
N1	0.63 (0.30–1.32)	0.223
NX	12.4 (0.26–586.57)	0.201
R		0.008
R1	1.07 (0.46–2.46)	0.877
R2	0.23 (0.00–10.83)	0.457
RX	2.72 (1.13–6.55)	0.025
High SIAH3	0.36 (0.15–0.85)	0.020

## Data Availability

Publicly available online data used for analyses are available via the cited sources. The RNA-sequencing of MIA-PaCa-2 and PANC-1 cell lines comparing gene expression in chronic exposure to 5% and 10% CO_2_ is available for download in Table S1.

## Supplementary Materials

The following supporting information can be downloaded at: https://www.mdpi.com/article/10.3390/ijms26072848/s1.

## Abbreviations

The following abbreviations are used in this manuscript:

ECM	Extracellular matrix
EMT	Epithelial-to-mesenchymal transition
GIST	Gastrointestinal stromal tumor
GSEA	Gene set enrichment analysis
HIF	Hypoxia-inducible factor
HUVEC	Human umbilical vein endothelial cells
PDAC	Pancreatic ductal adenocarcinoma
PDGF	Platelet-derived growth factor
SINA	Seven in absentia
SIAH	Seven-in-absentia homolog
TME	Tumor microenvironment
VEGF	Vascular endothelial growth factor
