# Supplementary material for: Gene Expression Profiling of Pancreatic Ductal Adenocarcinoma Cells in Hypercapnia Identifies SIAH3 as a Novel Prognostic Biomarker
Source: Int J Mol Sci. 2025 Mar 21;26(7):2848. doi: 10.3390/ijms26072848 (PMC11988995; doi:10.3390/ijms26072848)
Supplement: Supplementary file 1 [file ijms-26-02848-s001.zip › ijms-3414276-Supplementary Figures.pdf]

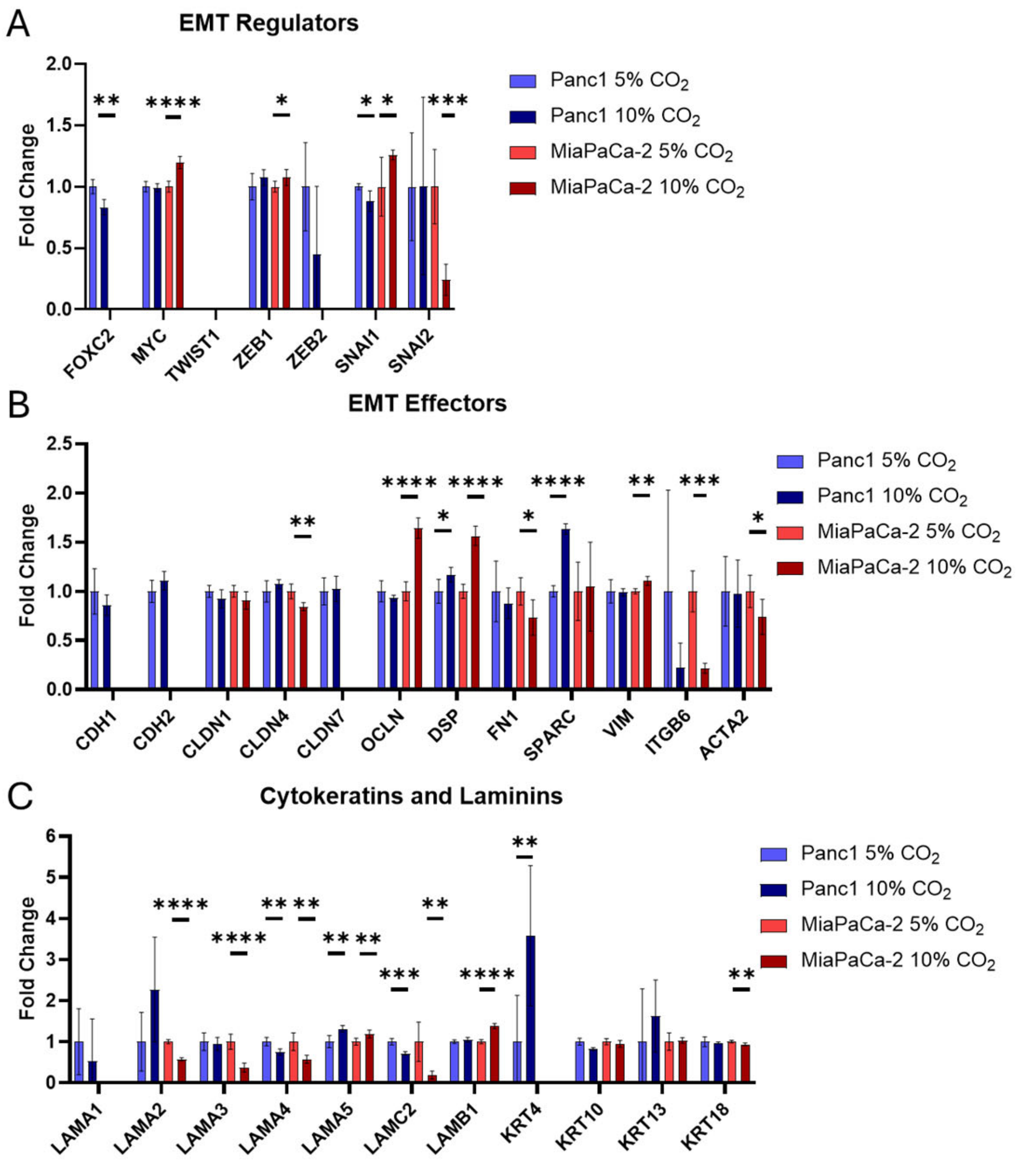

Supplementary Figure S1. RNA-Seq analysis of EMT associated targets in two pancreatic cancer cell lines (MiaPaCa-2, Panc1). (A) EMT regulators. (B) EMT effectors. (C) Cytokeratins and Laminins. Normalized count values were further normalized to 5% CO^2 arms. * P≤0.05, ** P≤0.01, *** P≤0.001, **** P≤0.0001.

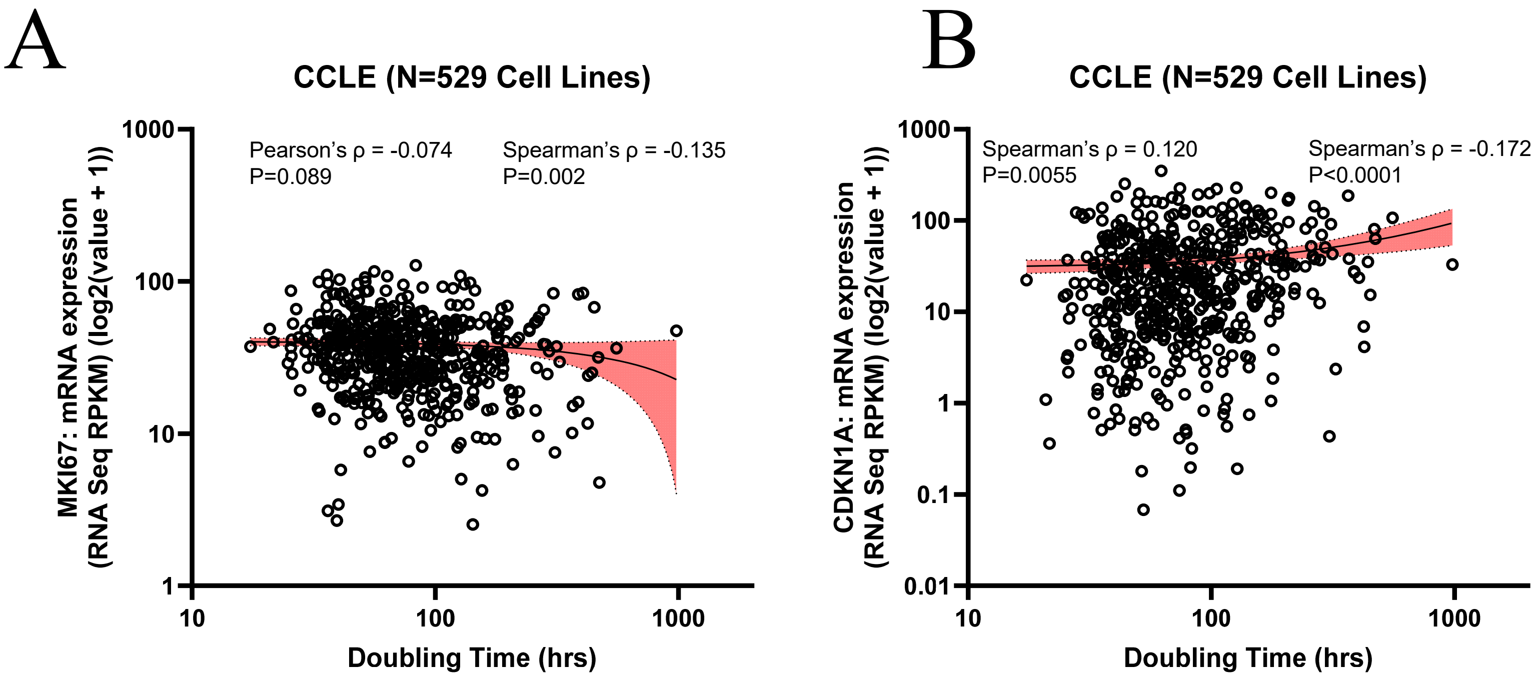

Supplementary Figure S2. (A) CCLE analysis correlation between MIK-67 RNA expression and doubling time in cancer cell lines (N=529). (B) CCLE analysis correlation between CDKN1A RNA expression and doubling time in cancer cell lines (N=529)

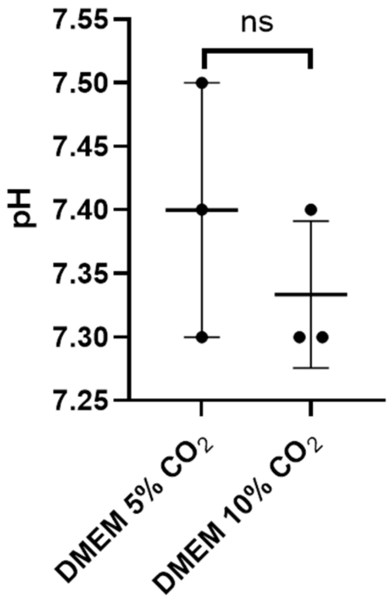

Supplementary Figure S3. DMEM Cell Culturing Media pH in 5% CO_2 and 10% CO_2
